# Key Positions of HIV-1 Env and Signatures of Vaccine Efficacy Show Gradual Reduction of Population Founder Effects at the Clade and Regional Levels

**DOI:** 10.1128/mBio.00126-20

**Published:** 2020-06-09

**Authors:** Changze Han, Jacklyn Johnson, Rentian Dong, Raghavendranath Kandula, Alexa Kort, Maria Wong, Tianbao Yang, Patrick J. Breheny, Grant D. Brown, Hillel Haim

**Affiliations:** a Department of Microbiology and Immunology, Carver College of Medicine, University of Iowa, Iowa City, Iowa, USA; b Department of Computer Science, University of Iowa, Iowa City, Iowa, USA; c Department of Biostatistics, University of Iowa, Iowa City, Iowa, USA; GSK Vaccines

**Keywords:** HIV-1, envelope glycoproteins, population-level evolution, vaccine design, virus diversity

## Abstract

HIV-1 group M was transmitted to humans nearly one century ago. The virus has since evolved to form distinct clades, which spread to different regions of the world. The envelope glycoproteins (Envs) of HIV-1 have rapidly diversified in all infected populations. We examined whether key antigenic sites of Env and signatures of vaccine efficacy are evolving toward similar or distinct structural forms in different populations worldwide. Patterns of amino acid variants that emerged at each position of Env were compared between diverse HIV-1 clades and isolates from different geographic regions. Interestingly, at each Env position, the amino acid in the clade ancestral or regional-founder virus was replaced by a unique frequency distribution (FD) of amino acids. FDs are highly conserved in populations from different regions worldwide and in paraphyletic and monophyletic subclade groups. Remarkably, founder effects of Env mutations at the clade and regional levels have gradually decreased during the pandemic by evolution of each site toward the unique combination of variants. Therefore, HIV-1 Env is evolving at a population level toward well-defined “target” states; these states are not specific amino acids but rather specific distributions of amino acid frequencies. Our findings reveal the powerful nature of the forces that guide evolution of Env and their conservation across different populations. Such forces have caused a gradual decrease in the interpopulation diversity of Env despite an increasing intrapopulation diversity.

## Abstract (precis)

The Env protein of HIV-1 is the primary target in AIDS vaccine design. Frequent mutations in the virus increase the number of Env forms in each population, limiting the efficacy of AIDS vaccines. Comparison of newly emerging forms in different populations showed that each position of Env is evolving toward a specific combination of amino acids. Similar changes are occurring in different HIV-1 subtypes and geographic regions toward the same position-specific combinations of amino acids, often from distinct ancestral sequences. The predictable nature of HIV-1 Env evolution, as shown here, provides a new framework for designing vaccines that are tailored to the unique combination of variants expected to emerge in each virus subtype and geographic region.

## INTRODUCTION

The founder of HIV-1 group M was transmitted to humans in the early part of the 20th century (1–3). Due to low fidelity of the viral replication machinery, frequent mutations are introduced in the HIV-1 genome (1, 2). The group M founder thus gradually diversified to create different genetic lineages (clades), which spread to multiple regions of the world (3, 4). In some regions, a single founder was introduced that accounts for most circulating strains, such as the clade B lineage in Korea (5) or the clade C lineage in India (6, 7). The envelope glycoproteins (Envs) on the surface of the virus are the most diverse of all proteins encoded by HIV-1; more than 20% of Env amino acids can differ between viruses from the same clade (8–11). This protein continues to diversify at a population level (11–14). Many epitopes recognized by broadly neutralizing antibodies (BNAbs) were intact in viruses that circulated during the early years of the pandemic but are now found in a significantly smaller proportion of strains (12). The antigenic diversity of Env poses a major challenge to the efficacy of vaccines (15–17). Several studies have examined the within-population changes that occurred in Env during the AIDS pandemic (11–14). However, less is known about the between-population changes. Are the same variants emerging in diverse clades and distinct geographic regions? Is Env evolving toward preferred structural forms? Are founder effects of the virus in diverse clades and newly infected regions stable over the course of time?

To address these questions, we examined the population-level changes that occurred in Env during the AIDS pandemic. We focused on two components of Env that contain multiple BNAb epitopes: (i) the glycan shield of gp120, composed of multiple N-linked glycosylation sites that adorn the surface of the molecule (18, 19); and (ii) the second variable loop (V2) segment at the trimer apex, which also contains two signatures of vaccine efficacy identified in sieve analysis of the RV144 trial results (20–23). Significant population-level changes have occurred during the pandemic in both components. Interestingly, from the clade ancestral or regional-founder virus, each position of Env has evolved toward a unique combination (frequency distribution [FD]) of amino acids that is highly conserved in different populations worldwide. FDs also exhibit clade-specific patterns; they are conserved in distinct geographic regions and monophyletic and paraphyletic subclade groups. For many Env positions targeted by BNAbs, founder effects of the virus at the clade and regional levels are gradually decreasing by evolution toward their site-specific FDs of amino acids.

## RESULTS

### Population-level changes in the glycan shield of Env are unique for each position and follow similar patterns in distinct regions worldwide.

**FIG 1:**
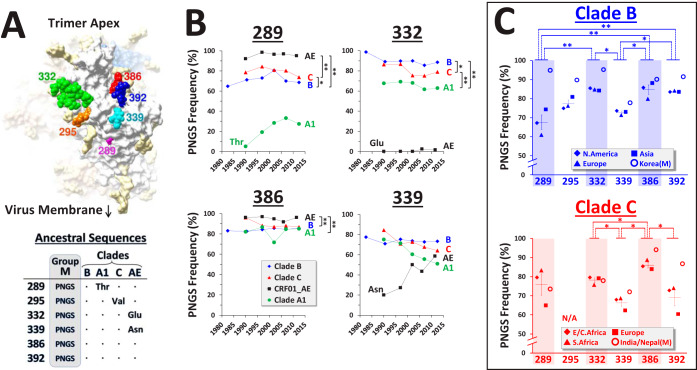
Population-level frequencies of PNGSs are specific for each position of Env and HIV-1 clade. (A) Cryo-electron microscopy image of the BG505 SOSIP.664 Env trimer (PDB ID 4TVP). Six sites occupied by a PNGS motif in the inferred group M ancestor are shown. Position 289 is occupied by threonine in the clade A1-derived BG505 Env. Ancestral sequences at these positions in four group M clades are shown below (dots denote presence of a PNGS motif). (B) Historical changes in PNGS frequencies at Env positions 289, 332, 386, and 339. Envs were isolated from samples collected worldwide between 1979 and 2015 (one sequence per patient). PNGS frequencies were calculated for consecutive 5- to 7-year periods in clades B (1,942 patients), C (1,248 patients), A1 (335 patients), and CRF01_AE (543 patients). The sequence variant found in the inferred ancestor of each clade (if not a PNGS motif) is indicated. A one-way analysis of variance (ANOVA) test was used to compare all time points between clades that contain a PNGS in their ancestral sequence. The following *P* values are indicated: *, *P* < 0.05; **, *P* < 0.01. (C) PNGS frequencies among recently circulating strains in different geographic regions (see year ranges and country compositions in Table S1). Averages and position specificity of the patterns (using a one-way ANOVA test) were calculated between regional panels that compose the paraphyletic groups of each clade. *, *P* < 0.05; **, *P* < 0.01. M, Envs from the monophyletic clusters that circulate in Korea and India/Nepal; NA, position not occupied by a PNGS in the clade ancestor. Error bars represent standard error of the mean (SEM).

We compared the historical changes in amino acid sequence at different sites of Env in diverse clades and geographic regions. Six adjacent positions on the Env trimer were first examined, which are occupied by the sequence motif for a potential N-linked glycosylation site (PNGS) in the group M ancestor (Fig. 1A). Glycans at these sites play critical roles in protecting Env from antibodies and paradoxically also serve as targets for some BNAbs (24–27). Historical changes in PNGS frequency at each site were examined in clades B, C, A1, and CRF01_AE, using 1,942, 1,248, 335, and 543 sequences, respectively, each from a different patient (see phylogenetic trees in Fig. S1 in the supplemental material). At many positions occupied by a PNGS in the clade ancestral sequences, frequency of this motif was relatively constant during the pandemic and specific for each clade (e.g., positions 289, 332, and 386 in Fig. 1B; see all six positions in Fig. S2A). Positions not occupied by a PNGS in the clade ancestors often showed considerable increases in frequency of this motif (e.g., position 339 in CRF01_AE).

10.1128/mBio.00126-20.1 FIG S1 Phylogenetic trees of Envs used in this study. Each Env is derived from a distinct patient. (A to C) Trees for clades B (1,942 patients), C (1,248 patients), and A1 (335 patients) were constructed by the maximum likelihood method using nucleotide sequences of the Env segment that spans the region from amino acid positions 131 to 511 (for clades B and C) or 156 to 459 (for clade A1). All trees are rooted to the clade ancestral sequences. (D) The tree for CRF01_AE (543 patients) was constructed using nucleotide sequences of the entire env gene. Amino acid alignments of all sequences included in this study are found at https://github.com/haimlab/HIV. Download FIG S1, PDF file, 1.7 MB. Copyright © 2020 Han et al. 2020 Han et al. This content is distributed under the terms of the Creative Commons Attribution 4.0 International license.

10.1128/mBio.00126-20.2 FIG S2 (A) Historical changes in PNGS frequencies at six positions of gp120 shown in Fig. 1B. PNGS frequencies are calculated in consecutive 5- to 7-year periods for each clade. The sequence variant in the inferred ancestor of each clade (if not a PNGS) is indicated. A one-way ANOVA test was used to compare all time points between clades that contain a PNGS in their ancestral sequence. The following *P* values are indicated: *, *P* < 0.05; **, *P* < 0.01. (B) Frequencies of PNGSs at six positions of gp120, calculated among strains recently circulating in the indicated regions (see year ranges for each group in Table S1). Values are shown only for regional panels that contain a PNGS motif at the indicated positions in their inferred ancestors. The clade B panel from Asia does not include samples from Korea. M, monophyletic clusters of clade B and C viruses from Korea and India/Nepal, respectively. Horizontal bars describe averages between regional panels of the paraphyletic groups of clades B and C and between both panels of CRF01_AE. Error bars represent SEM. Download FIG S2, PDF file, 0.5 MB. Copyright © 2020 Han et al. 2020 Han et al. This content is distributed under the terms of the Creative Commons Attribution 4.0 International license.

10.1128/mBio.00126-20.9 TABLE S1 Composition of HIV-1 Env panels used in this study. *^a^*M, number of sequences from the monophyletic clusters in Korea (clade B) and India/Nepal (clade C). Download Table S1, PDF file, 0.1 MB. Copyright © 2020 Han et al. 2020 Han et al. This content is distributed under the terms of the Creative Commons Attribution 4.0 International license.

To examine the position specificity of PNGS frequencies, we compared populations infected by the same HIV-1 clade from distinct geographic regions (Fig. 1C). We analyzed Envs isolated from recently collected samples (defined for most regional panels as the year range 2007 to 2015; see composition of all panels in Table S1). Within the clades, PNGS frequencies were specific for each position (see position specificity within clades B and C in Fig. 1C and comparison of the four clades in Fig. S2B). The range of frequency values was surprisingly narrow for the regional panels of the clade B paraphyletic group (from North America, Europe, and Asia): 84 to 85% at position 332, 73 to 74% at position 339, and 83 to 84% at position 392. The monophyletic cluster from Korea was introduced into this region in the late 1980s or early 1990s (5). Consequently, the frequency of PNGSs at all sites was higher in this lineage. Clade C also showed position-specific frequencies, which were similar in the regional panels of the paraphyletic group from Southern Africa (South Africa and Botswana), Eastern/Central (E/C) Africa, Europe, and also in the monophyletic cluster from India and Nepal (6, 7). Therefore, at six adjacent sites on the glycan shield of Env, the population-level frequencies of PNGSs are specific for each site and each clade. Within the paraphyletic groups of the clades, PNGS frequencies in populations from distinct regions occupy remarkably narrow ranges of values.

### Population-level frequency distributions of emerging variants are specific for each Env position and HIV-1 clade.

**FIG 2:**
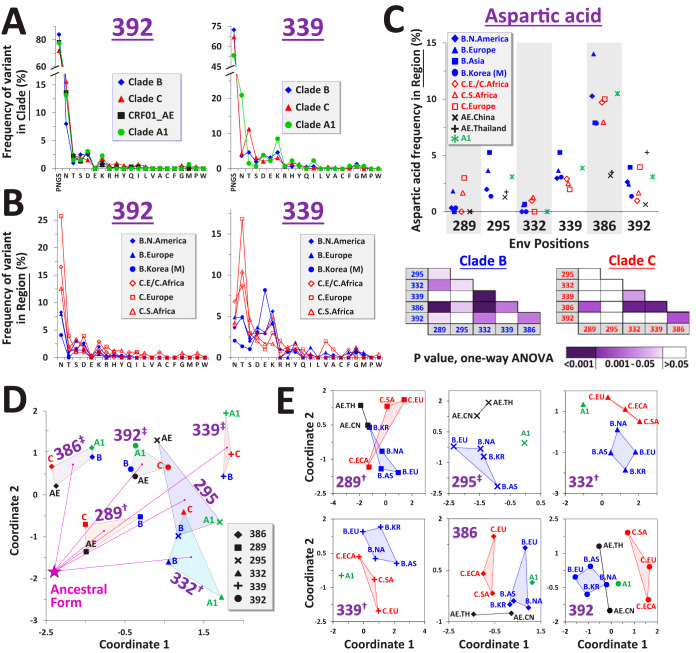
Frequency distributions (FDs) of amino acids that replaced the clade ancestral PNGS motif are specific for Env position and HIV-1 clade. (A) FDs at positions 392 and 339 in clades B, C, A1, and CRF01_AE, calculated among recently circulating strains. Clades that contain a PNGS motif at these positions in their ancestral sequence are shown. Residues are labeled by single-letter code. N, Asn that is not part of a PNGS motif. Profiles for all six positions are shown in Fig. S3A. (B) FDs at positions 392 and 339 calculated among recently circulating strains from the indicated regions (see also Fig. S3B to D). (C) Frequency of Asp in regional panels of clades B, C, A1, and CRF01_AE. Frequencies are shown for positions occupied by a PNGS motif in the clade ancestral sequences. A one-way ANOVA test was performed to compare frequencies between positions; cells are color-coded by *P* values. (D) Relationships between FDs in diverse clades. FD profiles are shown for clades that contain a PNGS motif at the indicated positions in their inferred ancestral sequence. Each data point represents a 21-feature vector that describes the frequency of all variants among recently circulating strains from the indicated clade. Location of a profile composed solely of PNGSs is labeled “Ancestral Form.” Dashed lines connect FDs for the same position, and a line is drawn from the ancestral form to the centroid of each. Position specificity of the patterns was calculated by a permutation test, based on distances between the 21-feature vectors. †, *P* < 0.05; ‡, *P* < 0.005. (E) Clade specificity of FDs shown in panel D. FDs were calculated among recently circulating strains from each region. Clade specificity of the patterns is indicated. †, *P* < 0.05; ‡, *P* < 0.005.

We examined whether the frequencies of amino acids that replaced the ancestral PNGS motif also show patterns of specificity for position and clade. The frequency distribution (FD) of amino acids at the first position of the PNGS triplet was compared between recently circulating strains from the diverse clades (see positions 392 and 339 in Fig. 2A and all six positions in Fig. S3A). At some positions (e.g., 392), similar frequencies of emerging variants were observed in the diverse clades, whereas other positions (e.g., 339) showed greater variation. Regional panels from the same clade presented similar patterns of the low-frequency variants (Fig. 2B and Fig. S3B to D). We analyzed the position specificity of frequency values for each amino acid (see Asp in Fig. 2C and other amino acids in Fig. S3F). Asp frequencies in the clade B regional panels occupied narrow ranges of values, which were specific for each position (see *P* values in the inset matrices). Clade C showed similar frequencies in Europe, Southern Africa, and E/C Africa. A comparable profile, albeit with greater variation, was observed for the smaller monophyletic clade C cluster from India and Nepal (Fig. S3D). The similar FDs observed in the monophyletic clusters and paraphyletic groups suggested that clade-specific patterns do not result from the mixing of viruses between populations. Furthermore, analysis of the clade ancestral nucleotide sequences at these sites showed that the specificity of the patterns cannot be attributed solely to differential synonymous codon usage (Fig. S3E).

10.1128/mBio.00126-20.3 FIG S3 Amino acid frequency distributions (FDs) at Env positions occupied by a PNGS motif in the ancestral sequences of clades B, C, A1, and CRF01_AE. (A) FD profiles for the indicated positions were calculated among viruses of each clade recently circulating worldwide. N, Asn that is not part of a PNGS motif. (B, C) FD profiles in regional panels of recently circulating strains from clades B and C. (D) FD profiles for clade C strains from Southern Africa and the monophyletic cluster from India and Nepal. (E) Nucleotide sequences in the inferred ancestors of the indicated clades at the first and third positions of the N-X-S/T triplet. Sites with identical nucleotide sequences are shaded similarly. Asterisks indicate absence of the sequence motif for a PNGS. Differential synonymous codon usage did not correspond with the patterns of emerging variants. For example, the inferred ancestors of clades B and A1 contain the same sequence (AAT/ACT) at positions 339/341 and 392/394. Nevertheless, the FDs that emerged at positions 339 and 392 are distinct (see Fig. 2D). (F) Frequencies of amino acids that replaced the PNGS motif in regional panels of Envs from clades B, C, A1, and CRF01_AE. Matrices above each figure show the results of a one-way ANOVA test that compares amino acid frequencies between positions within clades B and C. *P* values are color-coded as indicated on the right. Download FIG S3, PDF file, 0.3 MB. Copyright © 2020 Han et al. 2020 Han et al. This content is distributed under the terms of the Creative Commons Attribution 4.0 International license.

To determine position and clade specificity of the complete profile of all emerging variants at each position, we examined the relationships between FDs in diverse clades and geographic regions. For this purpose, the FD in each population was treated as a 21-feature vector that describes the log_10_ frequency of all 20 amino acids and a PNGS. Euclidean distances between vectors were calculated as a measure of differences between FDs. To visualize these relationships, the distance matrix between all vectors was used as input for multidimensional scaling (MDS), which scales this distribution down to two dimensions (28). We first examined position specificity of the FDs by comparing recently circulating strains from the four clades. FDs that evolved from a PNGS in the clade ancestors were compared. Clear clustering of the FDs for the same position was observed (see *P* values in Fig. 2D and approach to calculations in Materials and Methods). Some positions (e.g., 339) have diversified considerably (see lines between “Ancestral Form” that represents an FD composed only of PNGSs and the centroid of all clade FDs for each position), whereas others (e.g., 289) have diversified less. In addition to position specificity, a secondary pattern of clade specificity was observed (Fig. 2E). At positions 289, 295, 332, and 339, FDs of panels from the same clade but different regions were clustered (see *P* value labels in Fig. 2E). Clustering patterns were also observed at positions 386 and 392 but did not reach statistical significance. In addition, we analyzed the position specificity of FDs at the 13 sites of gp120 occupied by a PNGS motif in the ancestors of all four clades (Table S2). FDs exhibited various degrees of “migration” from the ancestral form and clear position-specific clustering patterns (Fig. S4).

10.1128/mBio.00126-20.4 FIG S4 Relationships between FDs at 13 Env positions occupied by a PNGS motif in the inferred ancestors of clades B, C, A1, and CRF01_AE. Data points represent FDs at the indicated positions calculated among recently circulating strains. FDs for the same position are connected by solid lines. Location of the ancestral state (a PNGS motif) is indicated by a star symbol. Position specificity of the patterns was calculated by a permutation test, based on distances between the 21-feature vectors. †, *P* < 0.05; ‡, *P* < 0.005. Download FIG S4, PDF file, 0.4 MB. Copyright © 2020 Han et al. 2020 Han et al. This content is distributed under the terms of the Creative Commons Attribution 4.0 International license.

10.1128/mBio.00126-20.10 TABLE S2 Inferred clade ancestral sequence at gp120 positions occupied by a PNGS motif in the group M ancestor. *^a^*Asterisk indicates presence of a PNGS at the position in the inferred clade ancestor. Download Table S2, PDF file, 0.1 MB. Copyright © 2020 Han et al. 2020 Han et al. This content is distributed under the terms of the Creative Commons Attribution 4.0 International license.

**FIG 3:**
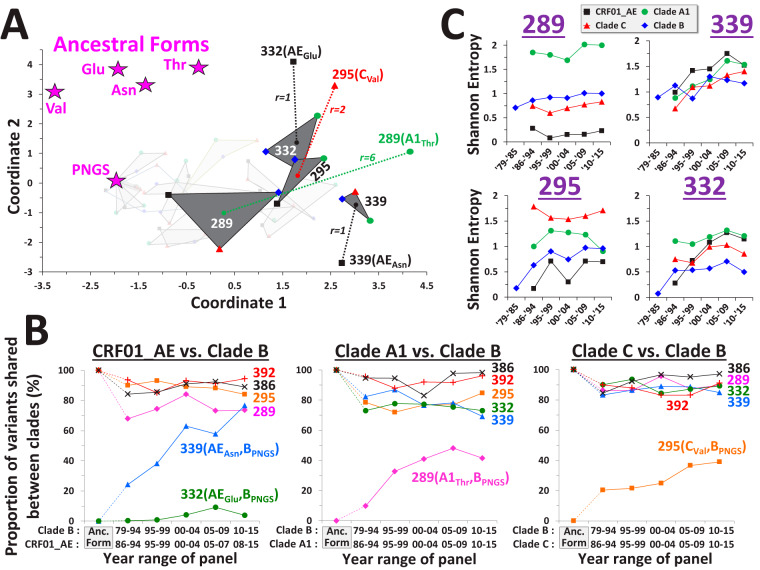
Env positions occupied by distinct variants in the ancestors of diverse clades have evolved toward similar FDs. (A) Distribution of FDs that evolved from distinct ancestral forms. Locations of the clade ancestral forms (i.e., FDs composed of a single sequence variant) are marked by star symbols. FDs that evolved from a clade ancestral PNGS are connected by gray triangles. FDs that did not evolve from a PNGS are labeled by the three-letter code of their ancestral form and connected by dashed lines to the centroid of the PNGS-derived FDs of the same position. Rank order of the distance to the corresponding centroid (relative to the centroids of the 17 positions of gp120 occupied by a PNGS motif in the group M ancestor) is indicated; *r* = 1 indicates that the centroid of the corresponding position is closest. FDs at 13 gp120 positions that evolved from a PNGS motif are shown in lighter shades for comparison (see also Fig. S4). (B) Historical changes in proportion of variants shared between diverse clades. Sum of the shared frequencies of all variants is shown (see also Fig. S5C). The sequence variants in the clade ancestors (if both not PNGSs) are indicated. Dotted lines mark changes from the clade ancestral forms. (C) Historical changes in Shannon entropy at the indicated positions in clades B, C, A1, and CRF01_AE.

We examined the FDs that emerged at the above positions when the clade ancestral form was not a PNGS motif (Fig. 3A and Fig. S5A). Position 339 in CRF01_AE evolved from an ancestral Asn (not part of a PNGS motif) to an FD similar to the PNGS-derived profiles of clades B, C, and A1 at this position. Of the 17 sites tested, the FD at position 339 in CRF01_AE was closest to the centroid of the PNGS-derived FDs of position 339 (i.e., ranked first in proximity; *r = *1 in Fig. 3A and Fig. S5B). Similarly, the FD at position 332 in CRF01_AE (derived from an ancestral Glu) was closest to the centroid of PNGS-derived FDs for position 332. Greater differences were observed between the PNGS-derived and non-PNGS-derived FDs for positions 295 and 289 (*r* = 2 and *r* = 6, respectively). These changes in FDs toward the common positional profile were associated with a gradual increase in the proportion of variants shared between clades (Fig. 3B and Fig. S5C). Changes in PNGS frequency at these sites accounted for much of the similarity (compare with Fig. 1B); however, other variants that replaced the ancestral forms also contributed. Importantly, for most positions, the decreasing interclade diversity occurred despite a gradual increase in the intraclade diversity (see Shannon entropy values in Fig. 3C).

10.1128/mBio.00126-20.5 FIG S5 Env positions evolved from distinct clade ancestral forms toward site-specific FD profiles. (A) FDs at Env positions 289, 332, 295, and 339. Values describe the frequency of variants among recently circulating strains isolated from samples collected worldwide. The sequence variant in each clade ancestor is indicated. (B) Relationships were analyzed between clade FDs at the 17 positions of gp120 occupied by a PNGS motif in the group M ancestor. The centroid of PNGS-derived FDs for the same position was calculated. Euclidean distances between the four FDs that are not derived from an ancestral PNGS and centroids of the 17 PNGS-derived FDs are shown. Distances are color-coded by value. The closest centroid to each non-PNGS-derived FD is highlighted by a red border. (C) Calculation of changes in proportion of shared variants between clades. As an example, variant frequencies at position 289 in clades B and A1 are analyzed for two time periods. The sum of the shared frequencies is indicated in the bottom graph. The line graph to the right shows the frequency of shared variants at all time periods. Download FIG S5, PDF file, 0.4 MB. Copyright © 2020 Han et al. 2020 Han et al. This content is distributed under the terms of the Creative Commons Attribution 4.0 International license.

Therefore, distinct Env positions that share the same sequence motif (a PNGS) in the clade ancestor have evolved unique FDs of emerging variants. FD profiles are specific for each position (Fig. 2D) and often for each clade (Fig. 2E). Some group M clade ancestors did not contain a PNGS motif at these sites (Fig. 1A). Such clade-founder effects have gradually decreased by evolution toward the position-specific FD profile. As a consequence, the interpopulation diversity at these sites has declined.

### Key positions in the trimer apex are evolving toward specific FDs and show a gradual reduction of clade- and regional-founder effects.

**FIG 4:**
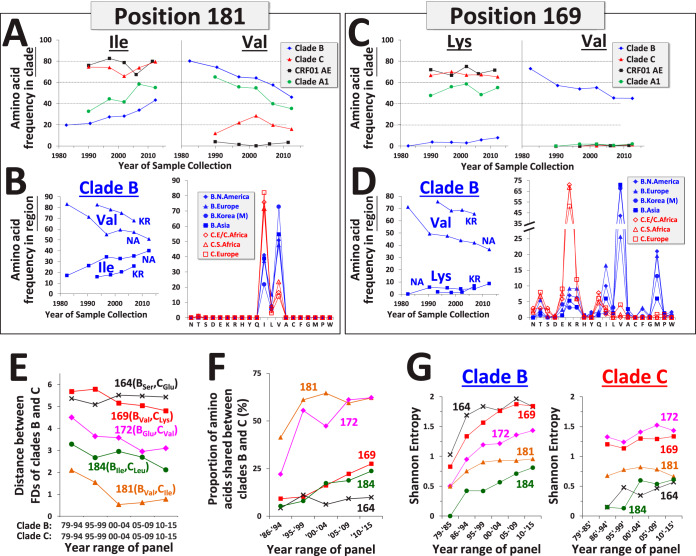
Signature sites of vaccine efficacy in the RV144 trial are evolving toward clade-specific FDs. (A, C) Historical changes in frequency of amino acids at positions 181 and 169 in diverse clades. (B, D) Changes in frequency of amino acids at positions 169 and 181 in clade B viruses from North America and the monophyletic cluster from Korea. Regional FDs among recent strains are shown to the right. FDs for all regional panels are shown in Fig. S6. (E) Historical changes in the distance between FDs of clades B and C. Positions in the V2 loop occupied by distinct amino acids in the clade B and C ancestors were analyzed. For each time period, the Euclidean distance between FD vectors of the clades was calculated. The amino acids in the clade ancestral sequences are indicated. (F) Historical changes in the proportion of amino acids shared between clades B and C at the positions shown in panel F. (G) Historical changes in Shannon entropy at the indicated positions in clades B and C.

The V2 variable loop segment at the apex of the Env trimer is targeted by several BNAbs (20–22). This domain has also been linked to vaccine efficacy in the RV144 trial. The presence of anti-V2 antibodies in vaccinated subjects was associated with lower infection rates (29). Furthermore, in breakthrough infection analyses, two signatures of vaccine protection have been identified, both located in the V2 apex (23). Vaccine efficacy was higher if the infecting strain contained Lys at position 169 or did not contain Ile at position 181. We examined amino acid occupancy at these sites in diverse clades and their evolution during the pandemic. At position 181, the ancestors of clades B and A1 contained Val, whereas the ancestors of clade C and CRF01_AE contained Ile. The frequency of Val in clades B and A1 rapidly decreased during the pandemic and was replaced by Ile (Fig. 4A). The monophyletic Korean cluster followed the same pattern but lagged behind the paraphyletic group. Among recently circulating strains, similar FDs were observed in the clade B panels (Fig. 4B and Fig. S6A). A comparison of FDs that emerged at position 181 with FDs at other positions occupied by Val in the clade B ancestor revealed the position-specific nature of each profile (Fig. S6C).

10.1128/mBio.00126-20.6 FIG S6 FDs of amino acids at positions 181 and 169 in regional panels of diverse clades. (A, B) Frequencies were calculated among recently circulating strains. Frequency values for Korea and India/Nepal were calculated among isolates of the monophyletic clusters that circulate in these regions. The clade B panel from Asia does not include isolates from Korea. The amino acid found in each clade ancestral sequence is labeled “Anc.” (C) Relationships between regional FDs at gp120 positions occupied by Val in the clade B ancestor (for North America, Europe, and Asia) and in the founder of the monophyletic cluster from Korea. Location of the ancestral/founder form is indicated by a star symbol. Position specificity of the patterns is shown. †, *P* < 0.05; ‡, *P* < 0.005. Download FIG S6, PDF file, 0.3 MB. Copyright © 2020 Han et al. 2020 Han et al. This content is distributed under the terms of the Creative Commons Attribution 4.0 International license.

At position 169, the inferred ancestors of clades A1, C, and CRF01_AE contained Lys (associated with protection), whereas the clade B ancestor contained Val (Fig. 4C). The frequency of Val gradually decreased in clade B to less than 50% of circulating strains and was replaced primarily by Met and Ile (Fig. 4D, Fig. S6B, and Fig. S7A). The same pattern of change was observed in the monophyletic lineage from Korea and the North American panel (Fig. 4D). Among recently circulating strains, similar FD profiles were observed for the different regional panels of clade B (Fig. 4D and Fig. S6B).

10.1128/mBio.00126-20.7 FIG S7 (A) Historical changes in frequency of amino acids at position 169 in clades B and C. (B) Historical changes in FDs at position 169 in clades B and C. Download FIG S7, PDF file, 0.6 MB. Copyright © 2020 Han et al. 2020 Han et al. This content is distributed under the terms of the Creative Commons Attribution 4.0 International license.

Comparison of the variants that emerged at positions 169 and 181 in clades B and C (right panels of Fig. 4B and D) suggested that a considerable proportion of amino acids are shared between currently circulating strains in the two clades. We analyzed the relationships between the complete FD profiles in clades B and C at different time periods during the pandemic. Positions in the V2 loop occupied by different amino acids in the ancestors of clades B and C were examined, focusing on sites minimally affected by insertions or deletions. For each position, we calculated the distance between FDs in the paraphyletic groups of the clades (Fig. 4E). For some positions (e.g., 181 and 172), the interclade distance gradually decreased, whereas others show less or no change (e.g., position 164). These patterns were associated with a gradual increase in the proportion of shared amino acids between the two clades at each position (Fig. 4F). In some cases, the degree of similarity appears to have stabilized, whereas in others, it continues to increase. Therefore, clade-founder effects have gradually decreased during the pandemic for each position at a different rate. The decline in the interclade diversity occurred despite a gradual increase in the intraclade diversity at these positions (Fig. 4G).

**FIG 5:**
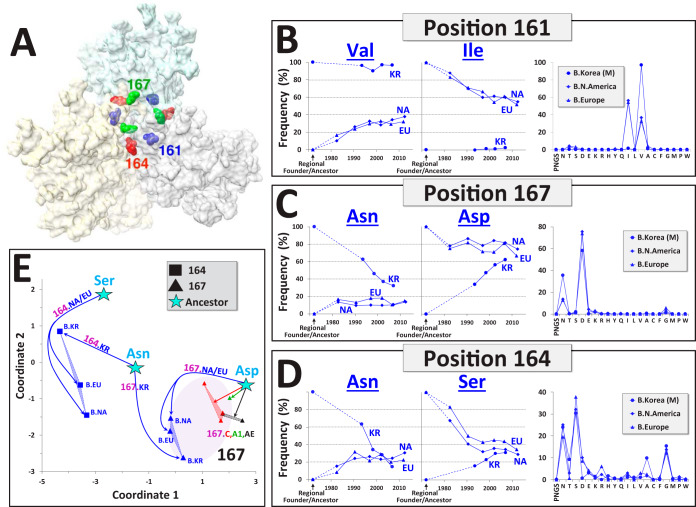
Key positions in the V2 apex have evolved from distinct amino acids in the clade B ancestor and Korean regional founder toward similar position-specific FDs. (A) Top view of the Env trimer apex (PDB ID 4TVP). Residues in the V2 loop that differ between the clade B ancestral sequence and the founder of the monophyletic cluster in Korea are labeled. (B to D) Historical changes in frequency of residues at the indicated positions in Korea, North America, and Europe. Dashed lines indicate the changes from the ancestral/founder form of each group. FDs among recently circulating strains in the three regions are shown to the right. (E) Relationships between clade B regional FDs at positions 164 and 167, calculated among recently circulating strains. Locations of the ancestral/founder forms are indicated by star symbols; solid lines are drawn to the FDs that emerged from each. Dotted lines connect between FDs of the same position and clade. FDs at position 167 in clades A1, C, and CRF01_AE are shown for comparison.

We sought to determine whether founder effects that occurred in a monophyletic lineage of the virus also decreased during the pandemic. Three differences in amino acid sequences were identified in the V2 segment of the trimer apex between the founder of the monophyletic clade B cluster in Korea and the ancestral sequence of the paraphyletic clade B group (see labeled positions in Fig. 5A). Significant changes occurred in FDs at these three positions during the pandemic (see Fig. 5B to D and FDs at all 30 positions in this segment in Fig. S8). At position 161, the Korean lineage retained the Val, whereas the frequency of this amino acid gradually increased in North America and Europe to replace the ancestral Ile (Fig. 5B). Consequently, at position 161, the paraphyletic group evolved toward a profile similar to that of the Korean lineage. At position 167, the major changes occurred in the Korean lineage, which rapidly evolved toward a profile similar to the paraphyletic group (Fig. 5C). At position 164, the ancestor of the clade B paraphyletic group contained Ser, whereas the Korean lineage founder contained Asn (Fig. 5D). In all panels, the frequency of Ser gradually evolved toward a value of 29 to 34% and the frequency of Asn toward a value of 20 to 30%. Similar FDs were observed at this position among recently circulating strains of the different regional panels. Indeed, a comparison of clade B regional FDs at positions 164 and 167 showed clear changes from the distinct ancestral/founder forms toward the position- and clade-specific FDs (Fig. 5E).

10.1128/mBio.00126-20.8 FIG S8 FDs of variants at positions 156 to 185 in the V2 loop of gp120 among recently circulating clade B strains from the indicated regions. Frequency values for Korea were calculated among isolates from the monophyletic cluster that circulates in this region. Download FIG S8, PDF file, 0.4 MB. Copyright © 2020 Han et al. 2020 Han et al. This content is distributed under the terms of the Creative Commons Attribution 4.0 International license.

These results demonstrate the conserved nature of the forces that have guided changes at each site of Env in different populations worldwide. Such forces are sufficiently strong to decrease founder effects at the clade and regional levels. Evolution of Env toward well-defined FDs resulted in a gradual and considerable decrease in the interpopulation diversity of this protein despite a gradual increase in the intrapopulation diversity.

## DISCUSSION

The Env protein of HIV-1 is tremendously diverse in each population it has infected worldwide (3, 4, 8). The wide range of circulating forms poses a significant challenge to the development of an effective vaccine. Several studies have shown that previously conserved epitopes on Env are gradually lost, each at a distinct rate (12–14). However, to our knowledge, there has been no report of any clear “directionality” to the changes at a population level (i.e., toward a specific structural form). Here, we show that HIV-1 does evolve at a population level toward defined “target” states; however, these states are not specific amino acids but rather specific distributions of amino acid frequencies. FDs are primarily specific for each position of the molecule, and they show a secondary level of specificity for each clade. Key antigenic sites and signatures of vaccine efficacy have rapidly changed during the pandemic toward well-defined FDs. Such changes have reduced founder effects of the virus at the clade and regional levels. The rapid decrease in founder effects is surprising but also provides opportunities for future design of AIDS vaccines.

The replication machinery of RNA viruses is prone to errors. Random mutation events continuously introduce changes in sequence. Persistence of each variant within the host is determined by constraints applied on RNA secondary structure and by selective pressures applied on the protein on fitness and sensitivity to the host immune response. Establishment of the persistent variants in the population is determined by the bottlenecks applied during transmission and conservation of all selective pressures among different hosts (30–33). Our findings suggest that the combined result of the stochastic events that generate the variants and the above deterministic forces is a conserved distribution of forms that is specific for each position. Such forces are sufficiently strong to produce similar profiles in distinct lineages of the virus, even for the lowest-frequency variants. Therefore, in contrast to the in-host environment, which is dominated by stochastic changes (12, 34, 35), the population-level distribution of circulating forms is controlled by conserved deterministic forces.

What are the selective forces that have guided these population-level changes? Fitness pressure is likely the primary mode of selection (36–38). As such, residue frequencies may describe their mean relative fitness in all structural “contexts” within a clade. Clade-specific patterns can thus reflect the unique structural properties of their Envs, which present specific fitness constraints (9, 10). Immune pressure applied by antibodies commonly elicited in the infected individual may also cause conserved population-level changes (39–41). For example, the rapid replacement of Asn at position 339 of CRF01_AE by a PNGS (Fig. 1B) may result from higher resistance of the latter to antibody neutralization (24, 42–44). As such, clade-specific FDs may also reflect the unique antigenicity profiles of their Envs (45). Analyses of the relative fitness and neutralization sensitivity of variants at evolving sites of Env will reveal the nature of the pressures that have caused the observed population-level changes.

HIV-1 has diversified from the group M founder virus to create distinct lineages. Within each, the virus has continued to change in sequence and antigenic properties (9–12). Here, we compare evolutionary patterns of Env between different populations. Clear changes occurred from distinct ancestral/founder forms toward similar distributions; at many positions, more than 50% of residues are now shared between clades. Therefore, diversity has increased at the within-population level, whereas it has decreased at the between-population level. In some cases, the frequencies of variants appear to have stabilized, such as the six adjacent sites of gp120 occupied by PNGSs (Fig. 1B) or position 164 in the V2 loop apex (Fig. 5D). In other cases, such as the signatures of vaccine efficacy, changes appear to progress at historically constant rates at the clade and regional levels (Fig. 4A to D). Current patterns are clearly affected by the time allowed for the changes to occur. For example, the monophyletic clade B lineage in Korea, which dates to the 1960s (5), was likely introduced into this region in the late 1980s or early 1990s. Accordingly, this lineage shows similar patterns of change that are delayed relative to other clade B groups (e.g., Fig. 4B and D).

At several positions located in relatively conserved domains of Env, only a minority of currently circulating strains contain the clade ancestral variant. Particularly significant changes have occurred in clade B at the trimer apex, which is targeted by multiple quaternary-specific BNAbs, including CAP256-VRC26, PG9, PG16, and PGT145 (46–50). Such patterns correspond with the declining breadth of these antibodies (12). Changes that follow founder events in newly infected regions reveal the “preferred” distributions of forms. Here, we focus on the clade B lineage in Korea. At some positions, viruses from the paraphyletic group rapidly gained the Korean founder residue (e.g., position 161) (Fig. 5B). At other positions, the opposite pattern was observed with rapid reduction of the founder effect (e.g., position 167) (Fig. 5C). Notably, some positions have evolved toward FDs that are not dominated by any single amino acid (e.g., position 164 in Fig. 5D or 170 and 183 in Fig. S8). Well-defined paths of change from the ancestral/founder form allow us to tailor immunogens to recently infected and poorly sampled populations according to the changes expected to occur toward the site-specific FDs at positions that compose key epitopes of Env.

## MATERIALS AND METHODS

### Analyses of HIV-1 Env sequences.

All HIV-1 *env* sequences were obtained from the Los Alamos National Lab (LANL) database using the sequence search interface (https://www.hiv.lanl.gov) and from the NCBI database (https://www.ncbi.nlm.nih.gov). Nonfunctional Envs were removed, as were sequences with nucleotide ambiguities or large deletions in conserved regions. A single *env* from each patient and a single sequence from known transmission pairs were used. In addition, a minimal nucleotide distance of 0.03 nucleotide substitutions per site was applied as a cutoff for selection. For phylogenetic analyses, nucleotide sequences were aligned using a Hidden Markov model with the HMMER3 software (51). Phylogenetic trees were reconstructed by the maximum likelihood method using PhyML3 (52). All Env positions described in the manuscript conform to the standard HXBc2 numbering of the Env protein (53). Potential N-linked glycosylation sites (PNGSs) were defined by the presence of the sequence Asn-*X*-Ser/Thr, where *X* can be any amino acid except Pro.

### Statistical analyses of frequency distributions and specificity of the patterns for position and clade.

Frequency distributions (FDs) describe the percent occupancy of Env positions by each variant relative to all variants in a defined population. Each FD is a vector composed of 20 or 21 features (20 amino acids and a PNGS). To calculate relationships between FDs, variants with frequencies lower than 0.75% (for regional panels) or lower than 0.6% (for whole-clade panels) were assigned a value of 0.1, and values were log_10_ transformed. Euclidean distances between all 21 feature vectors were then measured. For a graphical representation of the relationships between FDs, the distance matrix between their vectors was used as input for the Torgerson scaling method (28).

To determine the clade specificity of FDs, we first calculated for each position (p) the coordinates of the centroid ($C_{L}^{P}$) among vectors from the same clade (L). For each clade, the mean intraclade distance ($d_{\text{intraclade}}$) was calculated as the average Euclidean distance between $C_{P}^{L}$ and all regional vectors of the same clade ($R_{L}^{P}$), formally $\bar{\text{dist}(C_{L}^{P},R_{L}^{P})}$. In addition, we calculated the mean interclade distance ($d_{\text{interclade}}$) as the average Euclidean distance between the centroid of clade L and all other clade centroids $\overset{¯}{\text{dist}(C_{L}^{P},C_{L}^{P})}$ ∀ L′≠L. We define the ratio as $\frac{d_{\text{intraclade}}}{d_{\text{interclade}}}$. The baseline ratio (*S*_base_) was calculated as the ratio using all panels. Under the null assumption concerning the evolution of FDs, the intraclade distances are expected to be comparable to the interclade distances, while under the clade-specific alternative, we expect clustering of FDs within each clade even across regional populations. Therefore, within the position whose clade specificity is being calculated, clade identifiers were permuted and randomly assigned to each panel, from which the permuted ratio (*S*_rand_) was calculated. The permutation process was repeated 10,000 times. The *P* value was calculated as $\frac{\text{no. of times }S_{\text{rand}}\,<S_{\text{base}}}{10,000}$. To establish the position specificity of the profiles, the centroid of all regional profiles for a given position (*p*) was calculated ($C_{\text{All}}^{p}$). Here, intraposition distance ($d_{\text{intraposition}}$) was calculated as the average Euclidean distance between $C_{\text{All}}^{p}$ and all profiles of position p (for all clades and regions), more formally $\text{dist}(C_{\text{All}}^{P},R_{\text{All}}^{P})$ ∀ R, L, where $R_{L}^{P}$ denotes the profile in region *R* with position *p* and clade *L*. Then, interposition distance ($d_{\text{interposition}}$) was calculated as the average Euclidean distance between $C_{\text{All}}^{p}$ and all other positional centroids, more formally $\overset{¯}{\text{dist}\left(C_{\text{All}}^{p},C_{\text{All}}^{\mathrm{p\prime}}\right)}$ ∀ p′≠p. Finally, the ratio was determined as $\frac{d_{\text{intraposition}}}{d_{\text{interposition}}}$. Similar to clade specificity calculations, the baseline ratio (*S*_base_) was first calculated. Position identifiers were then permuted and randomly assigned to each panel, from which the permuted ratio (*S*_rand_) was calculated. The permutation process was repeated 10,000 times, and the *P* value was calculated as $\frac{\text{no. of times }S_{\text{rand}}<S_{\text{base}}}{10,000}$.

The source code for calculating FDs, position specificity, and clade specificity (including all data sets applied in this work) can be found at https://github.com/haimlab/HIV.
