# Supplementary figures and images for: Key Positions of HIV-1 Env and Signatures of Vaccine Efficacy Show Gradual Reduction of Population Founder Effects at the Clade and Regional Levels
Source: mBio. 2020 Jun 9;11(3):e00126-20. doi: 10.1128/mBio.00126-20 (PMC7373194; doi:10.1128/mBio.00126-20)

Figure S1

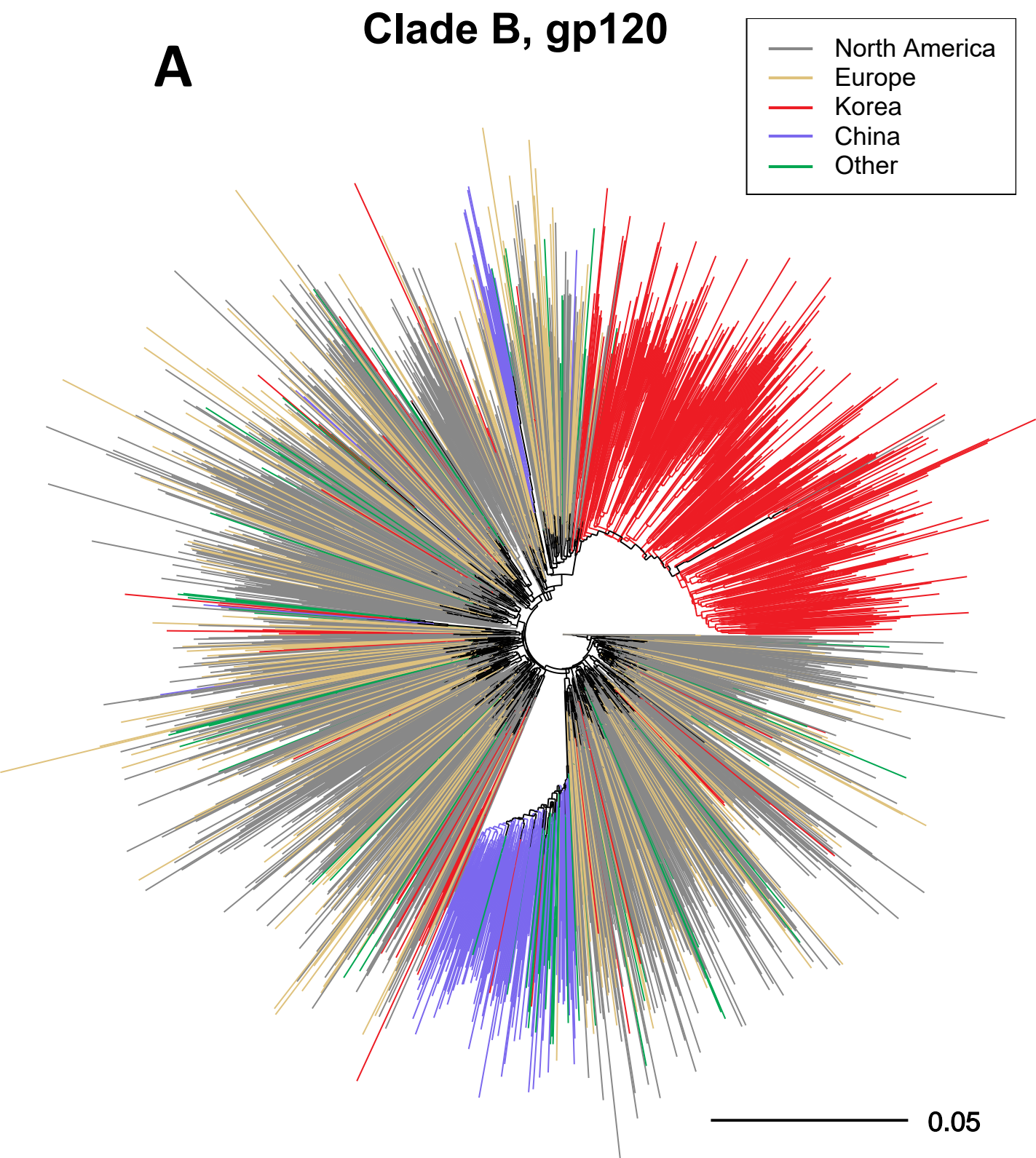

Figure S1

**B**

**Clade C, gp120**

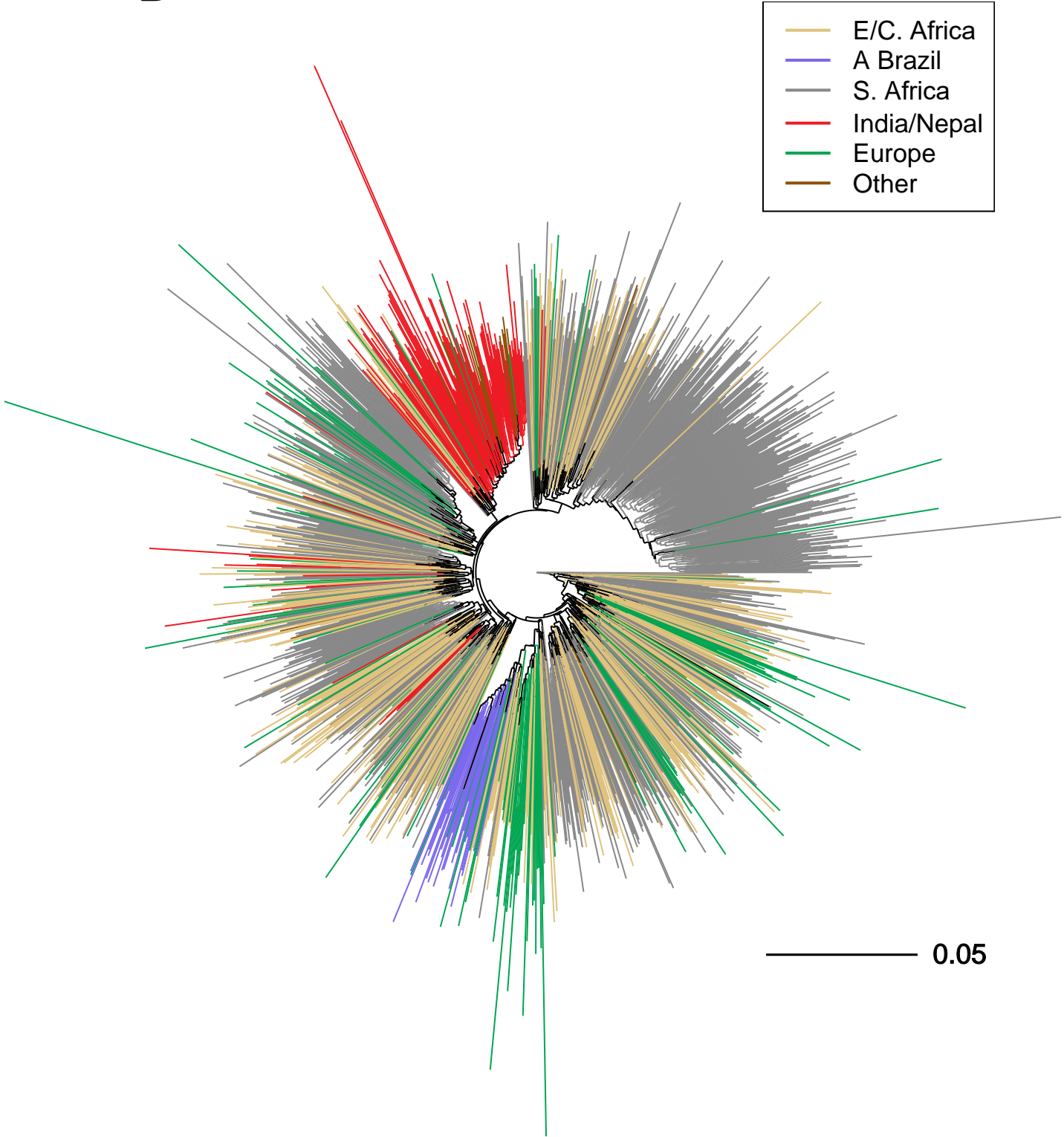

Figure S1

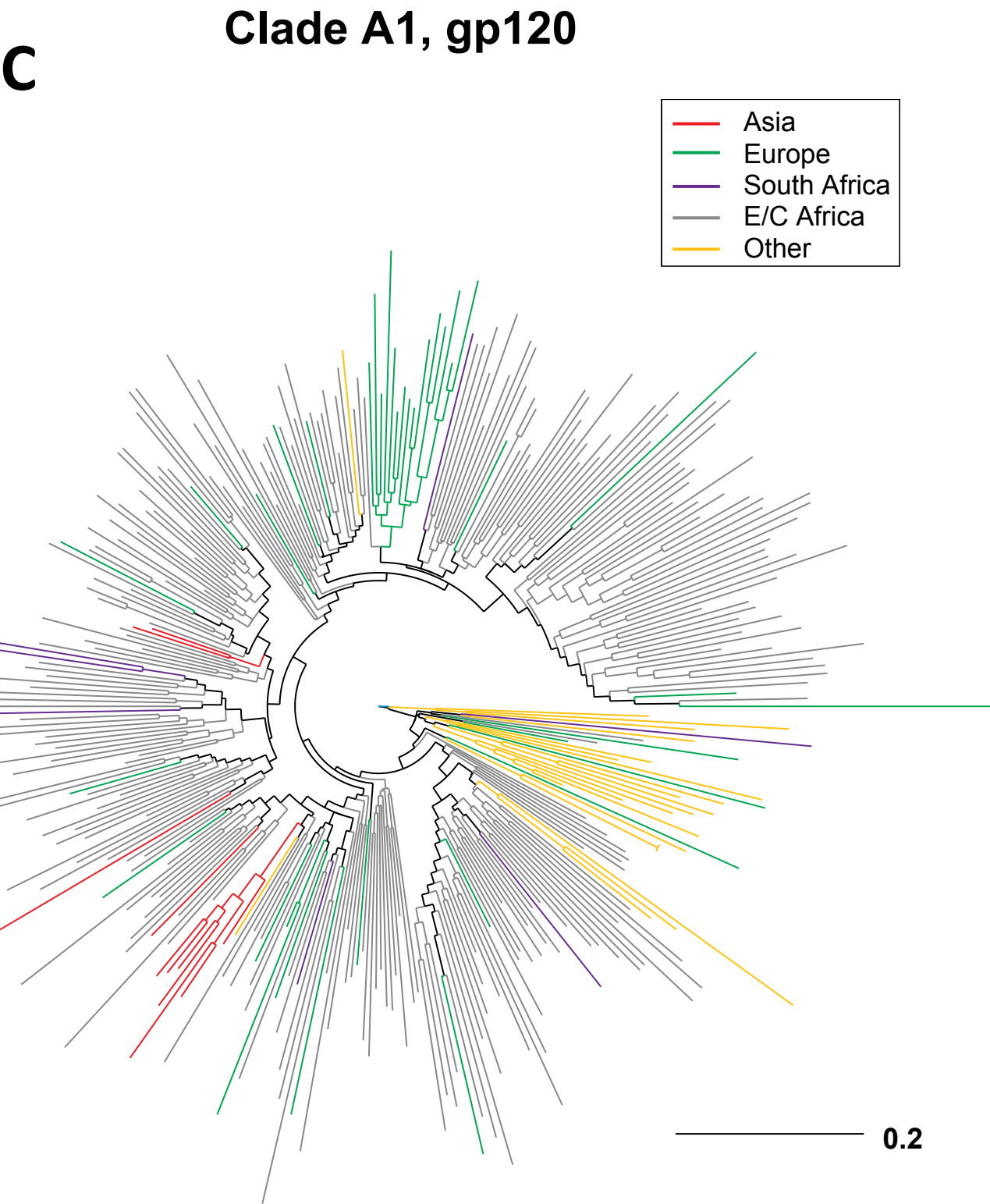

Figure S1

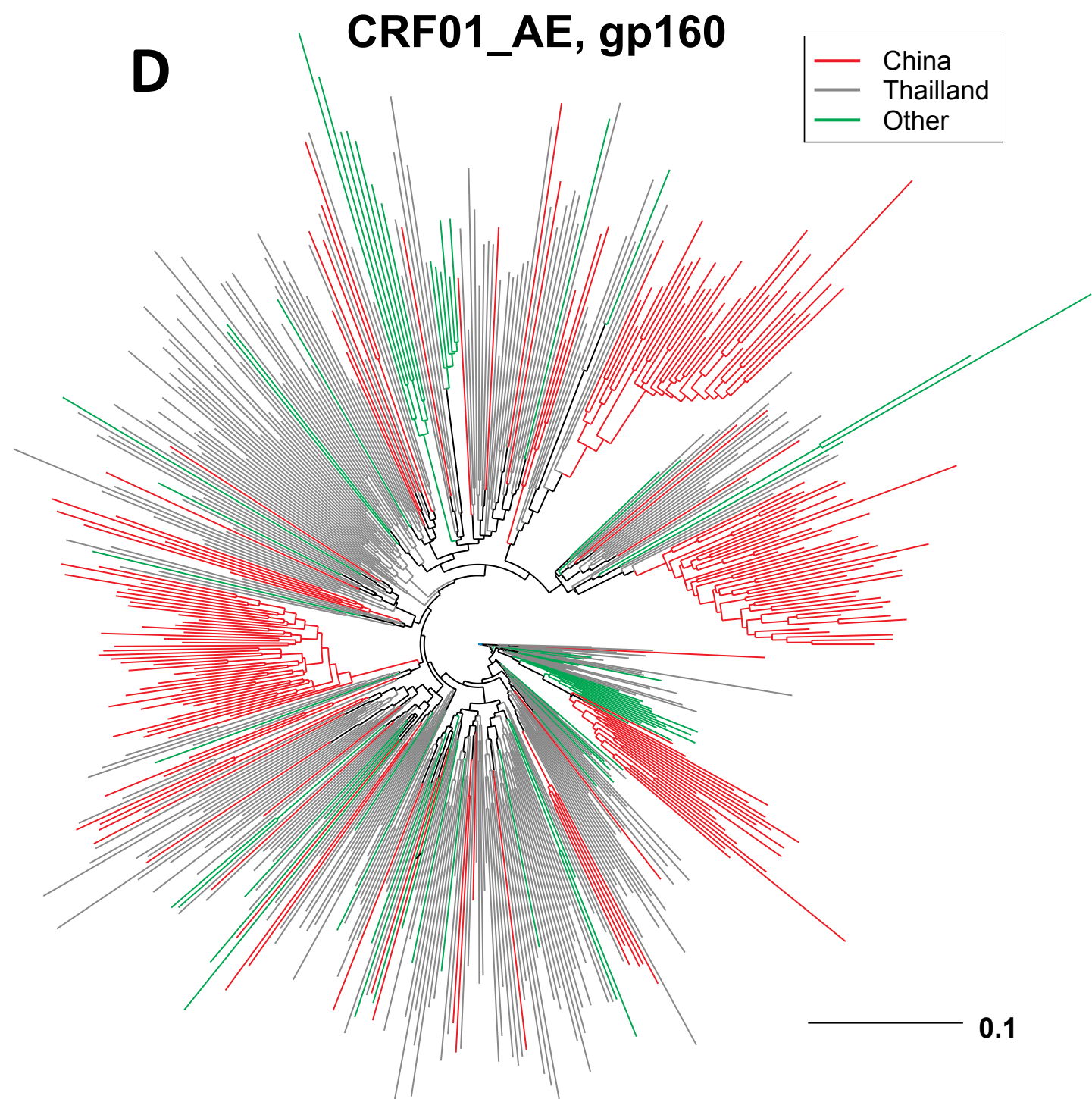

Supplement: FIG S1 [file mBio.00126-20-sf001.pdf]

Figure S2

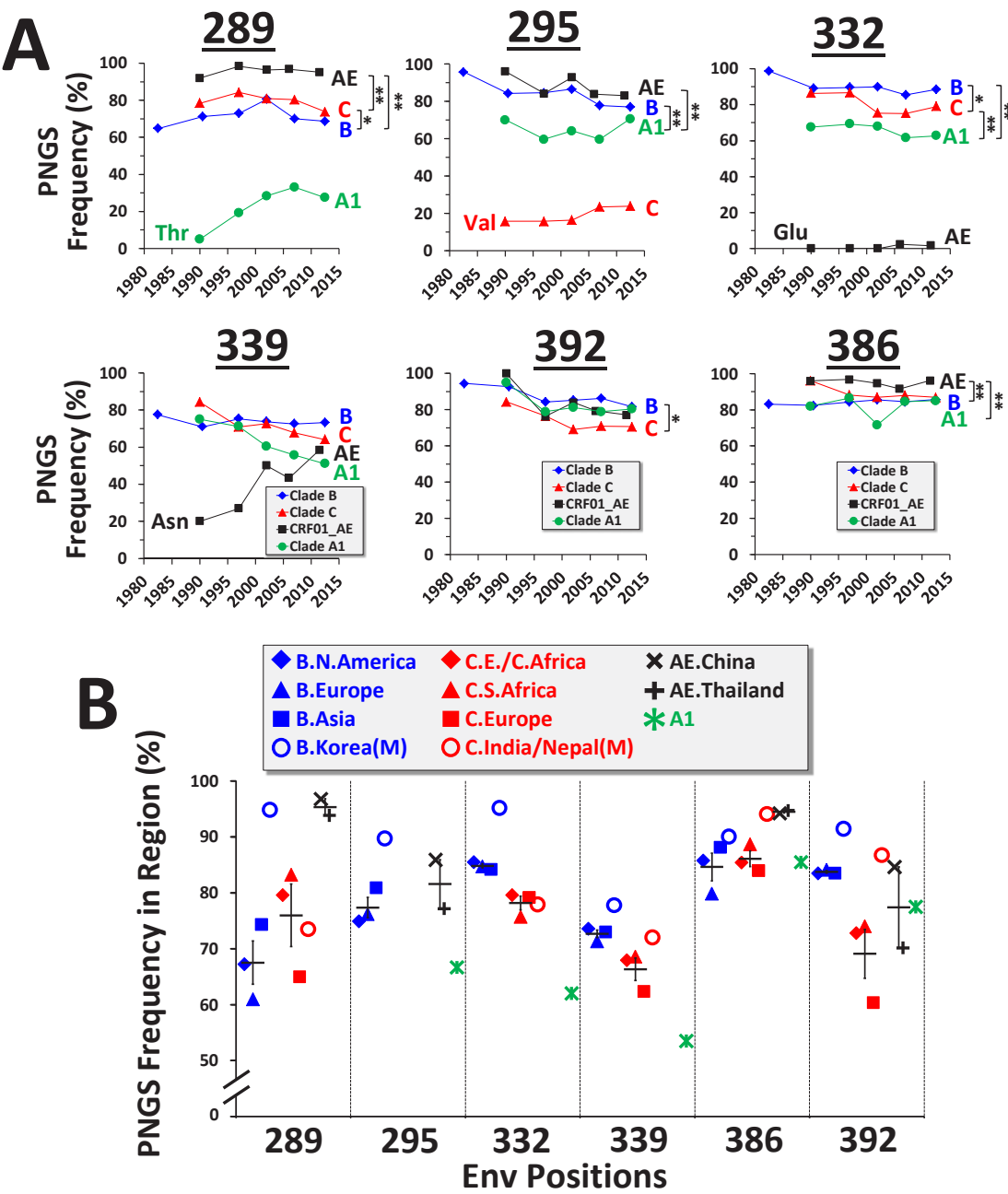

Supplement: FIG S2 [file mBio.00126-20-sf002.pdf]

## Frequency of variant in population (%)

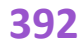

# E

Figure S3

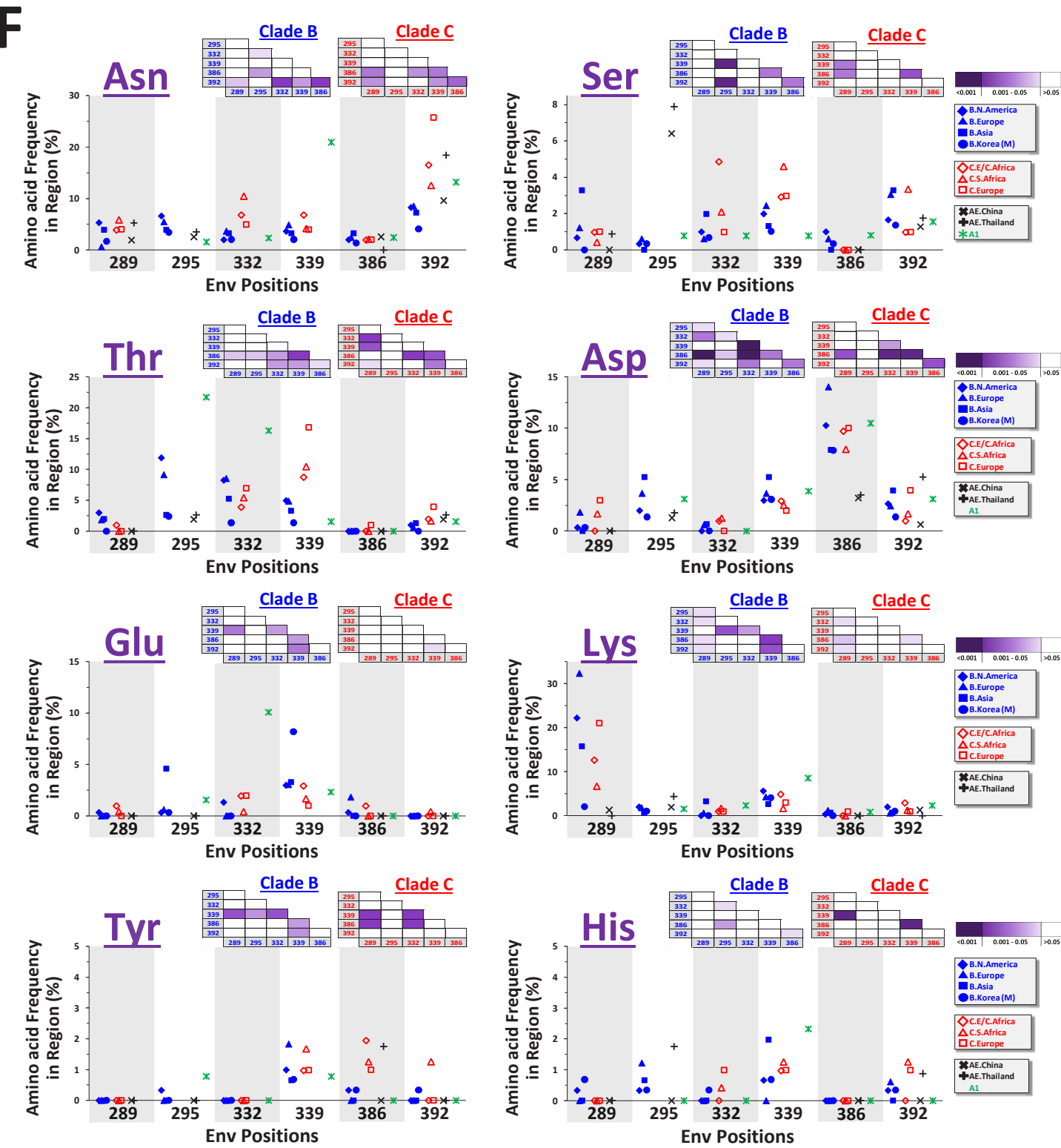

Supplement: FIG S3 [file mBio.00126-20-sf003.pdf]

Figure S4

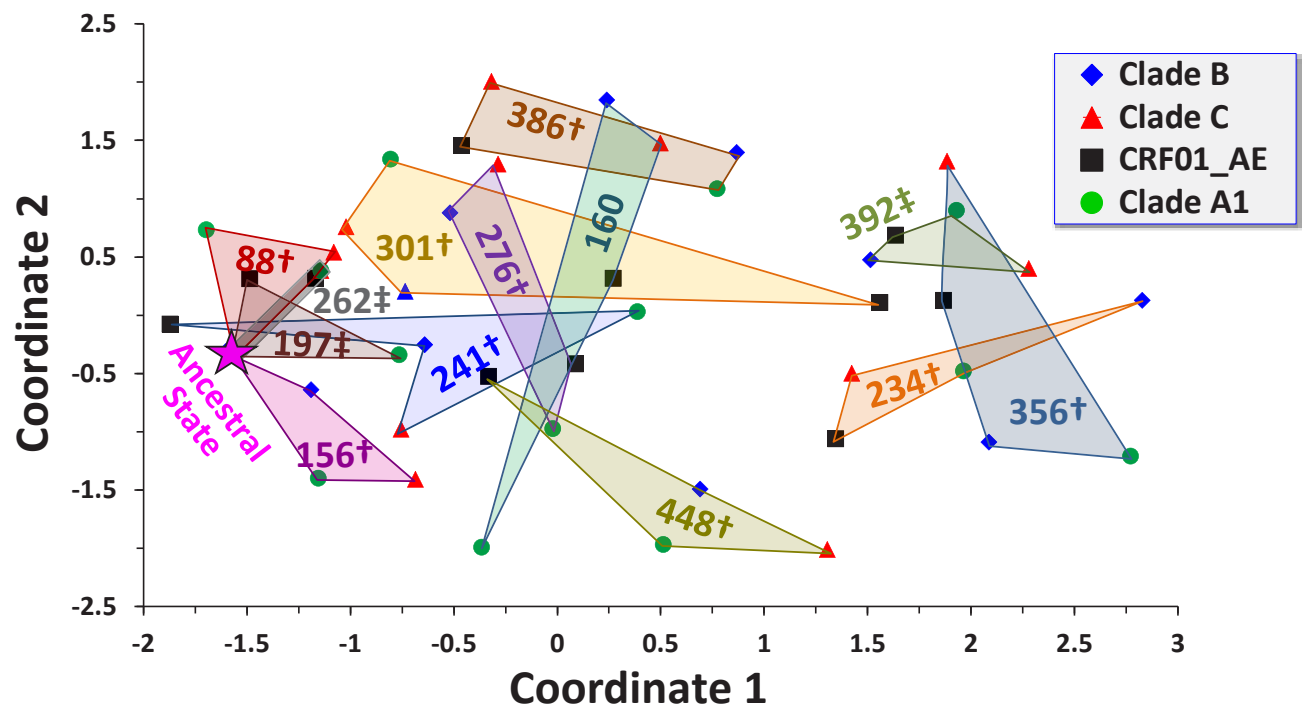

Supplement: FIG S4 [file mBio.00126-20-sf004.pdf]

Figure S5

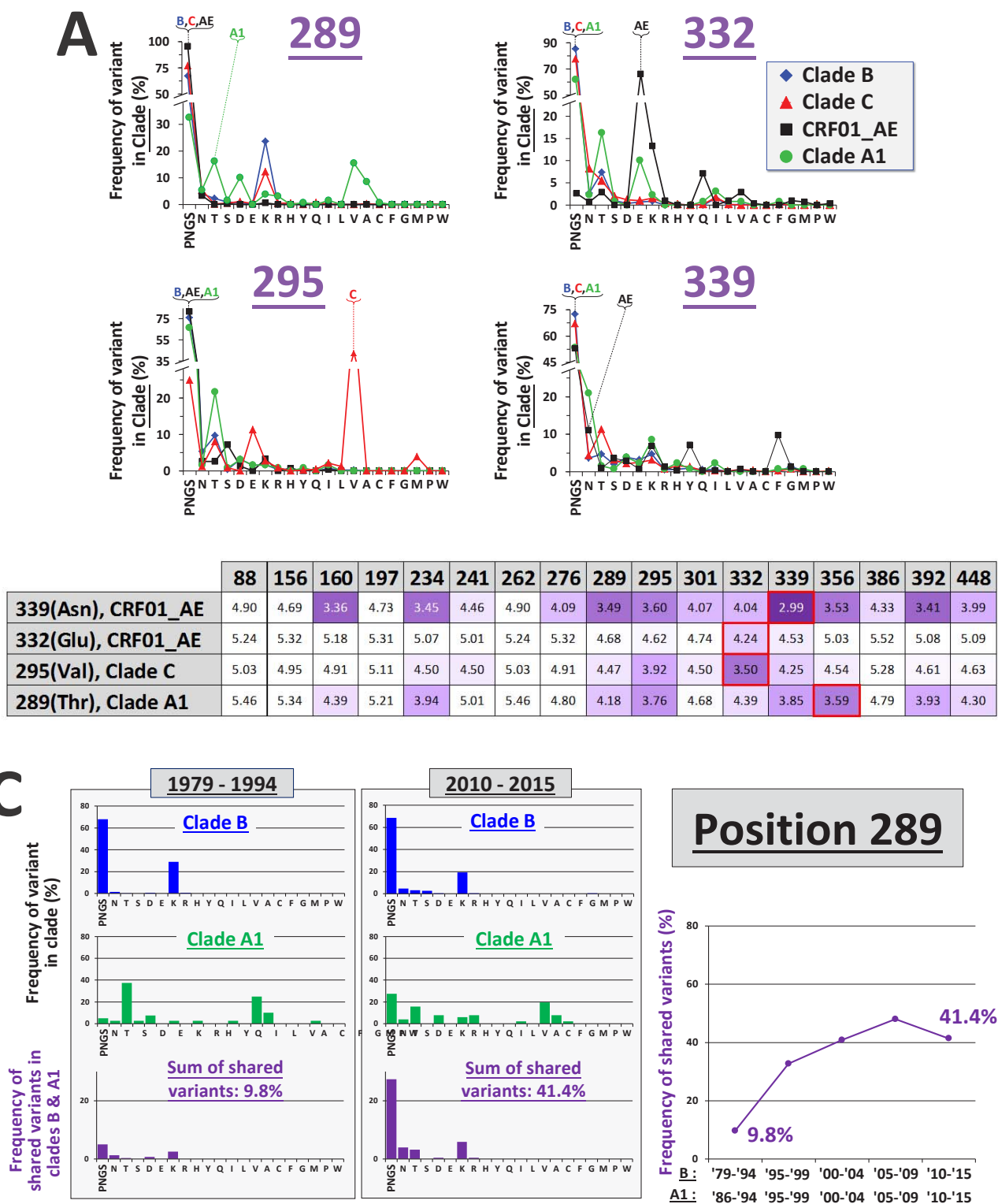

Supplement: FIG S5 [file mBio.00126-20-sf005.pdf]

Figure S6

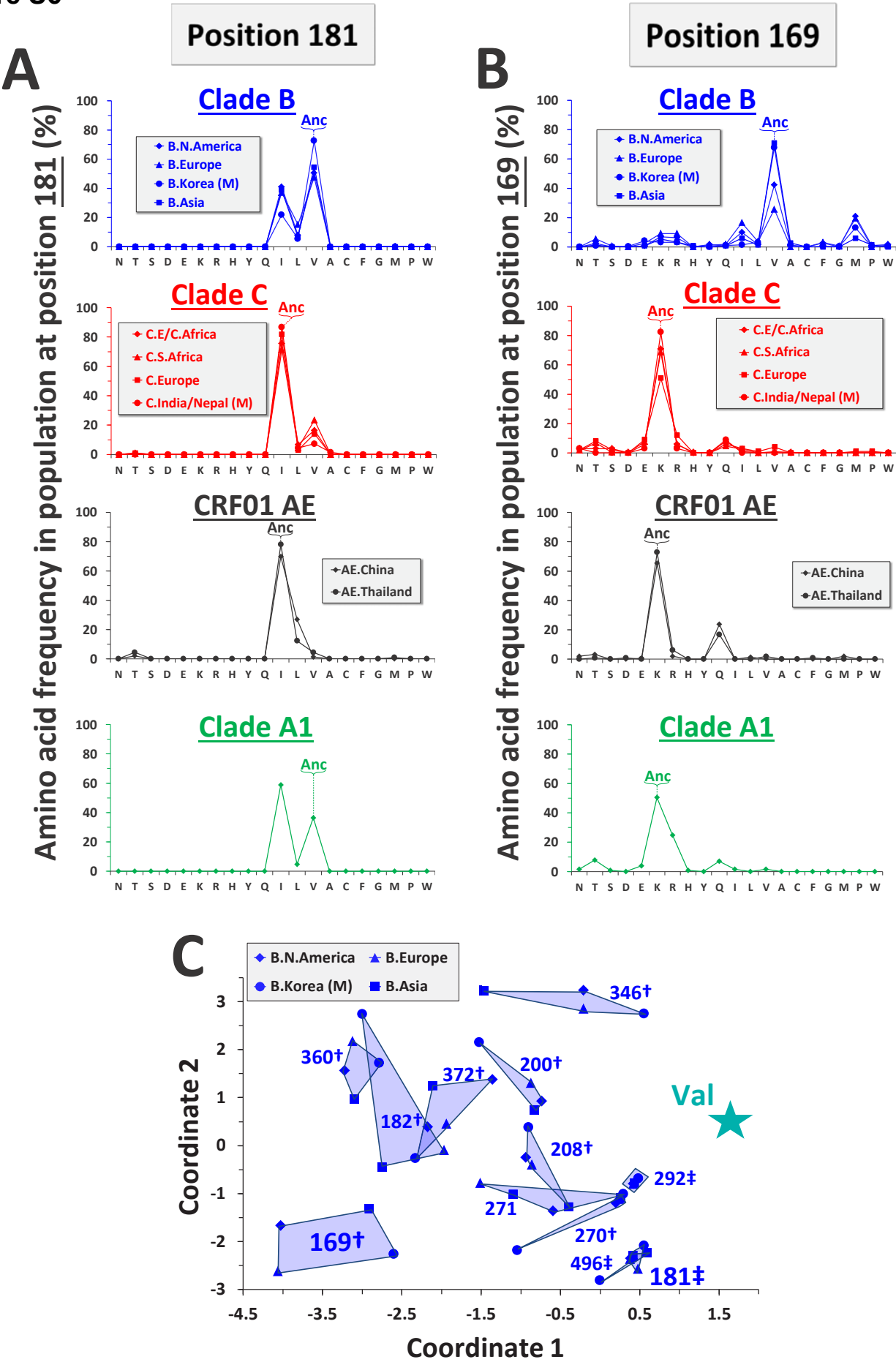

Supplement: FIG S6 [file mBio.00126-20-sf006.pdf]

Figure S7

A

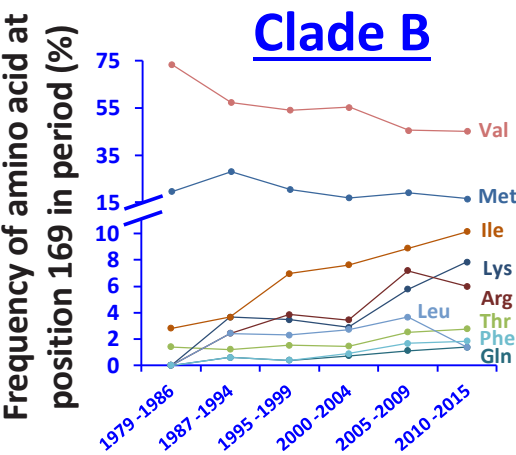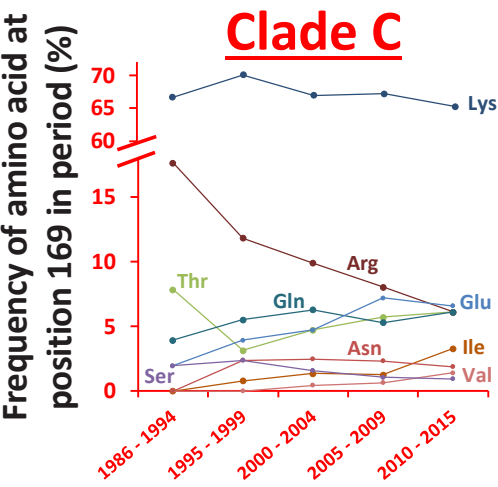

B

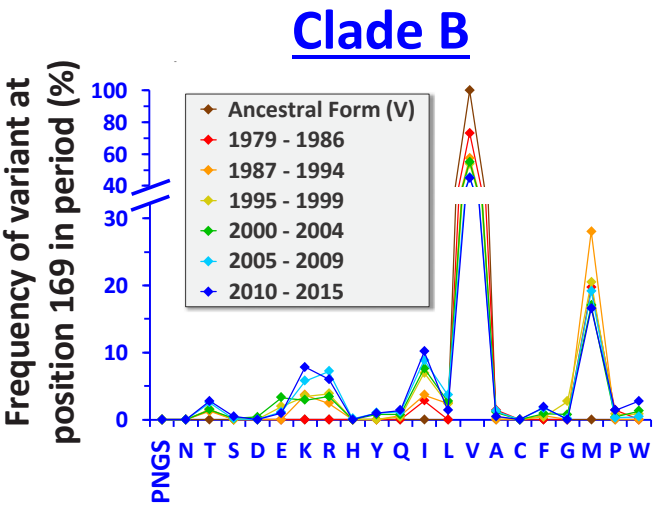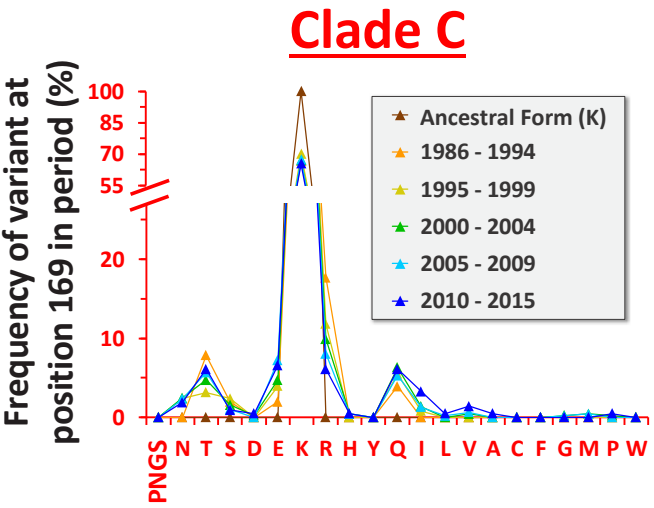

Supplement: FIG S7 [file mBio.00126-20-sf007.pdf]

Figure S8

Variant frequency in region (%)

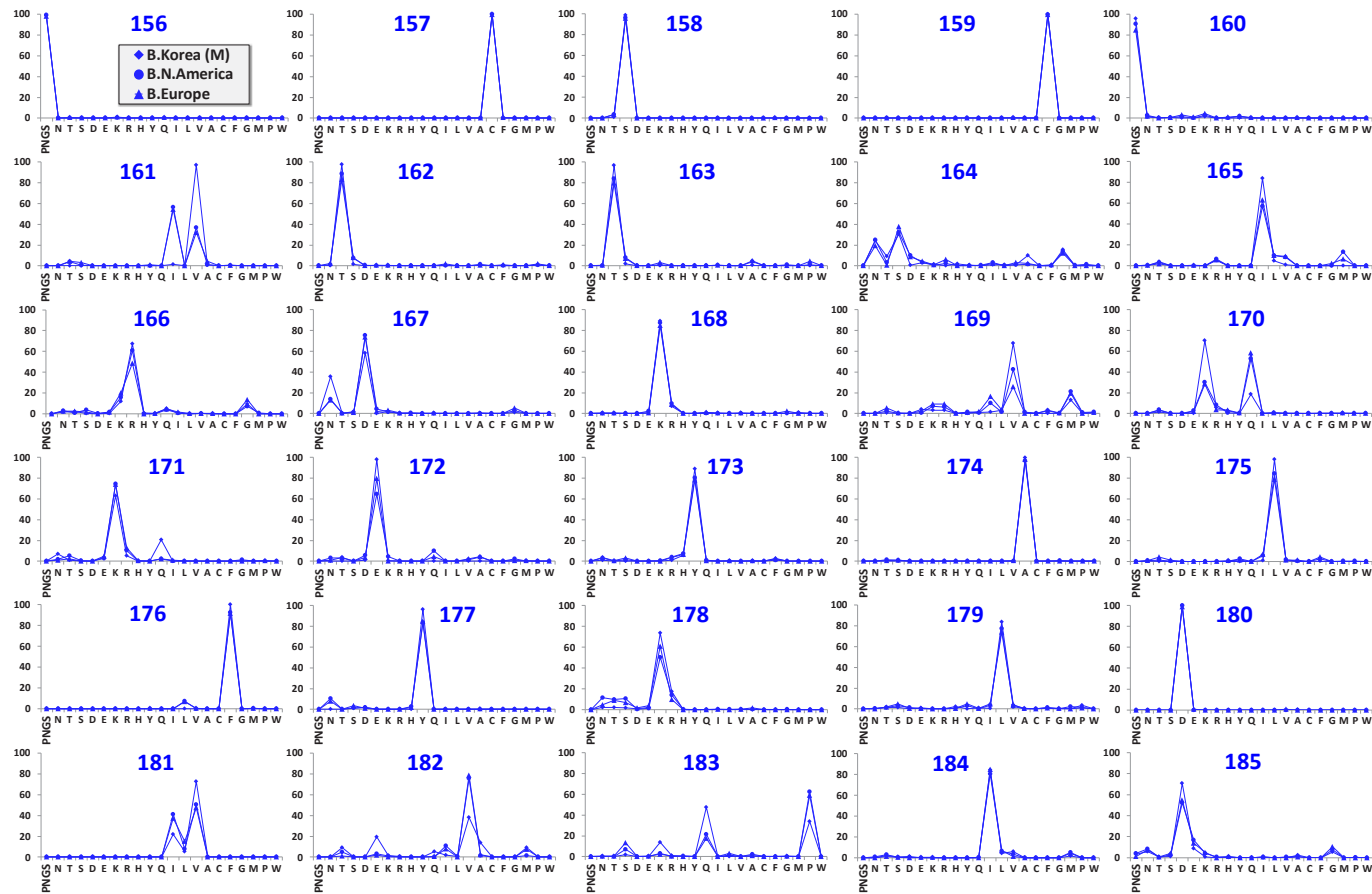

Supplement: FIG S8 [file mBio.00126-20-sf008.pdf]
