# Supplementary material for: Key Positions of HIV-1 Env and Signatures of Vaccine Efficacy Show Gradual Reduction of Population Founder Effects at the Clade and Regional Levels
Source: mBio. 2020 Jun 9;11(3):e00126-20. doi: 10.1128/mBio.00126-20 (PMC7373194; doi:10.1128/mBio.00126-20)
Supplement: TABLE S1 [file mBio.00126-20-st001.pdf]

Table S1. Composition of HIV-1 Env panels used in this study.

	Geographic Region	Number of Envs[a]	Countries	Tree Figure	Year range of 'current strain' group	Envs in each time period
**Clade B,** 1942 patients	North America (NA)	924	Canada, Cuba, Dominican Republic, Haiti, Jamaica, Trinidad Tobago, United States	Fig S1A	2007- 2015 (301)	1979-1986: 71<br>1987-1994: 155<br>1995-1999: 255<br>2000-2004: 535<br>2005-2009: 700<br>2010-2015: 217<br>2016 : 9
	Europe (EU)	330	Belgium, Switzerland, Cyprus, Germany, Denmark, Spain, France, Great Britain, Italy, Netherland, Sweden	Fig S1A	2007- 2015 (162)	
	Korea (KR)	474 (M=416)	S. Korea	Fig S1A	2001- 2009 (312, M=280)	
	Asia (AS)	212	China, Hong Kong, India, Japan, Myanmar, Philippines, Singapore, Thailand, Taiwan	Fig S1A	2005- 2015 (151)	
**Clade C,** 1248 patients	Southern Africa (SA)	657	South Africa, Botswana	Fig S1B	2007- 2015 (239)	1986-1994: 51<br>1995-1999: 125<br>2000-2004: 434<br>2005-2009: 464<br>2010-2015: 174
	Eastern/Central Africa (ECA)	348	Burundi, Djibouti, Ethiopia, Kenya, Malawi, Tanzania, Somalia, Uganda, Zambia	Fig S1B	2007- 2015 (103)	
	Europe (EU)	87	Belgium, Switzerland, Cyprus, Germany, Denmark, Spain, Finland, France, Great Britain, Italy, Netherlands, Portugal, Sweden	Fig S1B	2005- 2015 (64)	
	India/Nepal (IN/NP)	109 (M=94)	India, Nepal	Fig S1B	2000- 2015 (70, M=61)	
**CRF01_AE,** 543 patients	Thailand (TH)	283	Thailand	Fig S1C	2007- 2015 (114)	1986-1994: 25<br>1995-1999: 62<br>2000-2004: 56<br>2005-2007: 217<br>2008-2015: 183
	China (CN)	191	China	Fig S1C	2007- 2015 (156)	

**Clade A1,** 335 patients	All	340	Democratic Republic of Congo, Cameroon, Gambia, Kenya, Niger, Nigeria, Rwanda, Tanzania, Uganda, South Africa, Botswana, Belgium, Canada, Switzerland, Cyprus, Great Britain, Italy, Finland, India, Nepal, Korea, Pakistan, Sweden, United States	Fig S1D	2006- 2015 (122)	1985 : 1<br>1986-1994: 39<br>1995-1999: 51<br>2000-2004: 52<br>2005-2009: 145<br>2010-2015: 47

[a] M, number of sequences from the monophyletic clusters in Korea (clade B) and India/Nepal (clade C).
