# Supplementary material for: Key Positions of HIV-1 Env and Signatures of Vaccine Efficacy Show Gradual Reduction of Population Founder Effects at the Clade and Regional Levels
Source: mBio. 2020 Jun 9;11(3):e00126-20. doi: 10.1128/mBio.00126-20 (PMC7373194; doi:10.1128/mBio.00126-20)
Supplement: TABLE S2 [file mBio.00126-20-st002.pdf]

Table S2. Inferred ancestral sequences at gp120 positions occupied by a PNGS motif in the group M ancestor.

Env Region	Position	Clade-ancestral Sequence[a] B	C	01_AE	A1
C1	88	*	*	*	*
V1	156	*	*	*	*
V2	160	*	*	*	*
	197	*	*	*	*
C2	234	*	*	*	*
	241	*	*	*	*
	262	*	*	*	*
	276	*	*	*	*
	289	*	*	*	Thr
	295	*	Val	*	*
V3	301	*	*	*	*
C3	332	*	*	Glu	*
	339	*	*	Asn	*
	356	*	*	*	*
V4	386	*	*	*	*
	392	*	*	*	*
C4	448	*	*	*	*

[a] An asterisk indicates presence of a PNGS at the position in the inferred clade ancestor.
